# Blood Perfusion and Cellular Microstructural Changes Associated With Iron Deposition in Multiple Sclerosis Lesions

**DOI:** 10.3389/fneur.2019.00747

**Published:** 2019-07-11

**Authors:** Huaqiang Sheng, Bin Zhao, Yulin Ge

**Affiliations:** ^1^Department of Medical Imaging, Qianfoshan Hospital Affiliated to Shandong University, Jinan, China; ^2^Department of Radiology, New York University School of Medicine, New York, NY, United States; ^3^Department of Medical Imaging Research Institute, Shandong University, Jinan, China

**Keywords:** DTI (diffusion tensor imaging), susceptibility-weighted imaging, multiple sclerosis (MS), PWI = perfusion-weighted imaging, MRI - magnetic resonance imaging

## Abstract

**Background and Purpose:** Susceptibility-weighted imaging (SWI) has emerged as a useful clinical tool in many neurological diseases including multiple sclerosis (MS). This study aims to investigate the relationship between SWI signal changes due to iron deposition in MS lesions and tissue blood perfusion and microstructural abnormalities to better understand their underlying histopathologies.

**Methods:** Forty-six patients with relapsing remitting MS were recruited for this study. Conventional FLAIR, pre- and post-contrast T1-weighted imaging, SWI, diffusion tensor imaging (DTI), and dynamic susceptibility contrast (DSC) perfusion MRI were performed in these patients at 3T. The SWI was processed using both magnitude and phase information with one slice minimal intensity projection (mIP) and phase multiplication factor of 4. MS lesions were classified into 3 types based on their lesional signal appearance on SWI mIP relative to perilesional normal appearing white matter (peri-NAWM): Type-1: hypointense, Type-2: isointense, and Type-3: hyperintense lesions. The DTI and DSC MRI data were processed offline to generate DTI-derived mean diffusivity (MD) and fractional anisotropy (FA) maps, as well as DSC-derived cerebral blood flow (CBF) and cerebral blood volume (CBV) maps. Comparisons of diffusion and perfusion measurements between lesions and peri-NAWM, as well between different types of lesions, were performed.

**Results:** A total of 137 lesions were identified on FLAIR in these patients that include 40 Type-1, 46 Type-2, and 51 Type-3 lesions according to their SWI intensity relative to peri-NAWM. All lesion types showed significant higher MD and lower FA compared to their peri-NAWM (*P* < 0.0001). Compared to Type-1 lesions (likely represent iron deposition), Type-2 lesions had significantly higher MD and lower FA (*P* < 0.001) as well as lower perfusion measurements (*P* < 0.05), while Type 3 lesions had significantly higher perfusion (*P* < 0.001) and lower FA (*P* < 0.05). Compared to Type-2, Type-3 lesions had higher perfusion (*P* < 0.0001) and marginally higher MD and lower FA (*P* < 0.05).

**Conclusion:** The significant differences in diffusion and perfusion MRI metrics associated with MS lesions, that appear with different signal appearance on SWI, may help to identify the underlying destructive pathways of myelin and axons and their evolution related to inflammatory activities.

## Introduction

Multiple sclerosis (MS) is an inflammatory autoimmune neurodegenerative disease of the central nervous system (CNS), characterized by inflammation, demyelination, gliosis and neuro-axonal loss in lesions. It is generally believed that the basic pathogenesis of MS is collapse of immune tolerance to CNS myelin or myelin-like antigens followed by pro-inflammatory phagocytosis, oxidative injury, antigen presentation and T cell co-stimulation (1). Demyelinating and axonal injury are further consequences, which are typical features of MS. As we have already known, the progressive neurodegenerative processes in MS take a great toll on physical disability and cognitive disorder (2), and can seriously impact the quality of life in patients. Recent studies have shown that the changes of iron content that are commonly seen in MS lesions may be related to inflammatory activities (e.g., active myelin phagocytosis and intracellular iron depletion) and oxidative tissue injury in the demyelinating disease (3–6). Some other studies have found that iron is closely related to the biosynthetic enzymes of myelin formation (7, 8). Public opinions are divergent, but the effect of iron deposition on cellular and microstructural changes in the MS lesions remains an unresolved issue.

MRI has had an enormous impact on MS and plays a critical role as a paraclinical tool in routine clinical practice. The multi-sequence or multi-contrast MR imaging not only improves the diagnosis but also provides different specificity for various elements of pathology including iron deposition and microstructural destruction (9). Susceptibility weighted imaging (SWI) (10), as a three dimensional high resolution gradient echo sequence, is extensively applied for detecting abnormal iron deposition or microbleeds in MS (11). Compared to conventional T1- and T2-weighted MRI, SWI is more superior in displaying paramagnetic dark or hypointense signals, including the iron content in various forms of hemosiderin, ferritin and iron-laden macrophage (12–15) with high sensitivity even with only 1 gFe/g tissue iron changes (16). Studies have shown that MS lesions can also appear as an isointense or hyperintense signal on SWI with unclear pathophysiological implications (16, 17). It is therefore essential to identify the pathophysiological meaning of different SWI signal appearances of MS lesions using non-invasive imaging to fulfill this unmet need.

Recently, quantitative imaging measures have been increasingly used in MS research to better elucidate the hidden pathological mechanisms associated with tissue microstructural and inflammatory changes (9). Among these techniques, diffusion tensor imaging (DTI) (18–20) and dynamic susceptibility contrast MRI (DSC-MRI) (21, 22) are gaining more wide-spread utility in clinical practice and have shown great potential for detecting the cellular microstructural integrity and hemodynamic impairment at different stages of lesion evolution in MS, respectively. The aim of this study is to characterize the quantitative DTI-derived diffusion and DSC-derived perfusion parameters changes underlying different SWI signal intensities of MS lesions. We hypothesized that signal intensities detected on SWI in MS lesions may be a noninvasive biomarkers that can help clinicians to determine specific pathological processes associated with demyelination, axonal loss, and inflammatory processes in patients with relapsing-remitting MS.

## Materials and Methods

### Subjects

The research protocol of this retrospective study followed the tenets of the Declaration of Helsinki and was approved by the New York University Langone Health (NYULH) Institutional Review Board. Forty-six clinically definite relapsing remitting MS patients (28 women, 18 men, mean age 35.9 ± 11.3 years) enrolled from January 2012 to December 2016, were used in this study. All patients were informed and signed the institutional review board approved written consent form. The median disease duration in these patients was 4.4 years (range 1.6–11.4 years) and the median expanded disability status scale (EDSS) was 3.5 (range 1.5–5.5). These patients had no history of cerebrovascular disease, evidence of small vessel ischemic disease and no substantial intracranial pathology besides MS lesions in MR imaging.

### Image Acquisition and Processing

All patient data were acquired on a 3.0T Trio (Siemens Medical Solutions, Erlangen, Germany) MR scanner using a 20-channel array head coil. The MRI protocol included the following sequences: (1) Fluid-attenuated inversion recovery (FLAIR) imaging (TR/TE = 9420/134 ms, voxel size = 1 × 1 × 3 mm^3^); (2) pre and post T1-weighted (T1W) imaging (TR/TE = 630/15 ms, voxel size = 1 × 1 × 3 mm3); (3) susceptibility weighted imaging (SWI) (TR/TE = 28/20 ms; FA = 15°, voxel size = 0.86 × 0.86 × 3 mm^3^); (4) DTI with 30 directions (TR/TE = 7300/89 ms, voxel size = 3.0 × 3.0 × 3.0 mm^3^, b = 1000 s/mm^2^); (5) dynamic susceptibility contrast (DSC) perfusion imaging (TR/TE = 956/32 ms, voxel size = 1.7 × 1.7 × 3.0 mm^3^) applied to 13 axial slices centered at lateral ventricle body with 10 seconds injection delay. For DSC, a 3–5 cc/sec bolus of Gadolinium contrast agent (Gd-DTPA; Magnevist, Bayer Schering Pharma) was administered at a dose of 10–20 cc (0.075 mmol/kg) to acquire 60 time points. The post-contrast T1-weighted imaging (the same sequence with pre-contrast) was performed 10 min after injection. The image slice thickness from all sequences above is the same for lesion identification and registration on different imaging contrast. All sequences had 45 slices (13.5 cm) coverage of brain except DSC. The total scan time for all sequences was about 45 min.

SWI data is processed using an in-house image-processing software (SPIN) (23). The raw magnitude and phase from each SWI scan used to generate minimal intensity projection (mIP) using phase multiplication factor of 4 to enhance the susceptibility effects. Instead of using multiple slices for mIP, one slice mIP was used in this study to keep the slice thickness the same with the rest sequences and to minimize the partial volume effects from multi-slice mIP. DTI data analysis was performed offline using DTI studio, by which tensor images were generated to construct mean diffusivity (MD) and fractional anisotropy (FA) (24). MD and FA are the scalar measures of the total diffusion (e.g., average of eigenvalues) within a voxel and the degree of anisotropy in a given voxel, respectively. DSC data was processed using the perfusion analysis software package in Olea Sphere (Olea Medical, Cambridge, MA). Data first underwent preprocessing consisting of motion correction followed by spatial and temporal filtering. The standard single value decomposition (SVD) technique was then applied to the preprocessed data to generate maps of mean transit time (MTT), CBF, and leakage-corrected CBV (25). Because the absolute values of CBF (ml/100 ml/min) and CBV (ml/100 ml) can only be determined up to a multiplicative constant, the comparisons between lesion types were used as relative measures (i.e., rCBF, rCBV) in this study. Lastly, the diffusion and perfusion maps were manually registered to their corresponding conventional T1 and FLAIR imaging as well as SWI images using tkregister2 (Free Surfer, Massachusetts General Hospital, Harvard Medical School) for manually ROI placement and analysis.

### Data and Statistical Analysis

**Figure 1:**
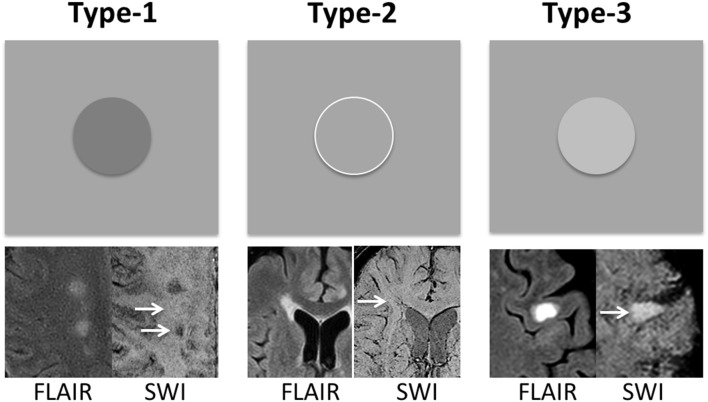
MS lesions are classified into three types based on their signal intensity appearance on SWI mIP image—Type-1: hypointense lesions; Type-2: isointense lesions; and Type-3: hyperintense lesions. The group classification is defined as the difference between lesion signal intensity and surrounding perilesional NAWM (peri-NAWM) is equal or over 30% cut-off. The arrows indicate the lesion types with different signal appearance on single-slice mIP SWI.

As shown in Figure 1, according to signal intensity appearances on SWI mIP, MS lesions were classified into three distinct lesion types. Type-1: hypointense (i.e., higher susceptibility), Type-2: isointense, and Type-3: hyperintense lesions. To avoid the visual predisposition bias, a cut-off value of 30% difference of mean intensity, measured between lesions and perilesional region, was applied. Only lesions with a diameter of 5 mm or larger were included in the data analyses. These lesions were first blindly reviewed and classified by each of the two experienced radiologists, and finally determined by consensus between the two for lesions with inconsistent opinion. Quantitative data analyses of diffusion and perfusion measurements were performed with Image J (National Institutes of Health, Bethesda, MD) software. Lesions were identified on conventional FLAIR, T1-weighted, and SWI images, on which the anatomical regions of interest (ROIs) were manually selected and then transferred onto co-registered FA, MD, CBF, and CBV maps. For each lesion, the ROI was placed on both lesion and perilesional NAWM (peri-NAMW) region for comparison. In order to increase the accurate lesion selection and avoid partial volume, the image with the lesion target was zoomed-in 3 times bigger on ImageJ for better ROI placement. On this magnified view, the ROI placement of peri-NAWM was also improved. Mixed model analysis of covariance (ANCOVA) was used to compare the lesions of each type to the perilesional normal appearing white matter (peri-NAWM) and to compare lesions of different types to each other with respect to FA, MD, rCBF, and rCBV. A separate univariate analysis was conducted for each perfusion measure. When the value of *P* < 0.05, the difference is considered to be statistically significant.

## Results

A total of 137 lesions were identified on conventional T2-weighted and post-contrast T1-weighted imaging in 46 patients with relapsing remitting MS that had both DTI and DSC data. Among them, there were 40 (or 29.2%) Type-1, 46 (or 33.6%) Type-2, and 51 (or 37.2%) Type-3 lesions (Figure 1). In addition, there were 11 enhancing lesions found in 6 patients; and 9 of these enhancing lesions were Type-3 lesions that showed hyperintensity on SWI, and another 3 enhancing lesions were Type-2 lesions that show isointensity on SWI. In contrast, none of the Type-1 lesions (hypointense on SWI) showed Gadolinium enhancement.

**Figure 2:**
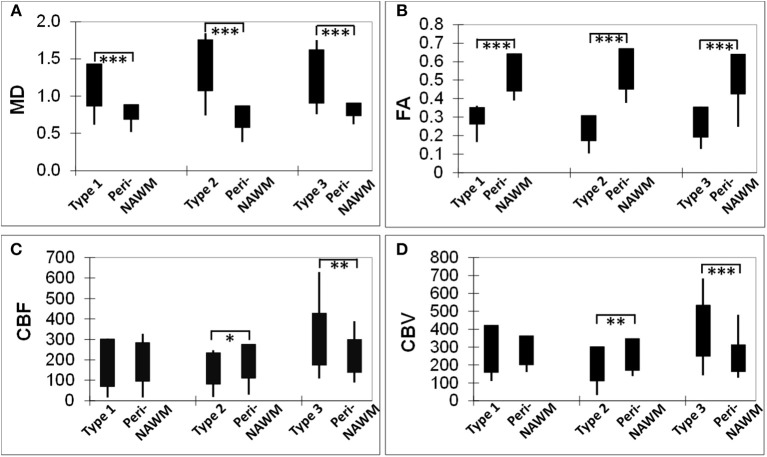
Bar graphs showing comparisons of DTI-derived MD **(A)** and FA **(B)**, as well as DSC-derived CBF **(C)** and CBV **(D)** measurements, between each lesion type and its corresponding perilesional normal appearing white matter (per-NAWM). ^*^P < 0.05, ^**^P < 0.01, ^***^P < 0.001. MD, CBF, and CBV are in their units of mm^2^/s, ml/100 g tissue/min, and ml/100 g tissue, respectively.

As shown in Figure 2, compared to peri-NAWM measurements, FA was significantly lower and MD was significantly higher in all types of lesions (*P* < 0.0001), indicating clear microstructural disruption in MS lesions. The mean FA values of Type-1, Type-2, and Type-3 lesions were 0.31 ± 0.05, 0.24 ± 0.07, 0.27 ± 0.08, respectively; and the mean FA values for their corresponding peri-NAWM were 0.49 ± 0.11, 0.52 ± 0.09, 0.45 ± 0.12 respectively. The mean MD values of Type-1, Type-2, and Type-3 lesions were 1.16 ± 0.27, 1.42 ± 0.34, 1.27 ± 0.36, respectively; and the mean MD values for their corresponding peri-NAWM were 0.71 ± 0.16, 0.68 ± 0.17, 0.82 ± 0.09, respectively. For perfusion measures, both CBF and CBV in Type-3 lesions (297.6 ± 126.5 ml/100 g/min, 385.9 ± 142.9 ml/100 g) showed significantly higher than peri-NAWM (216.9 ± 80.6 ml/100 g/min, 234.7 ± 75.6 ml/100 g) with *P* = 0.0002 and *P* < 0.0001, respectively. Type 2 lesions showed significantly lower CBF than peri-NAWM (158.6 ± 77.1 vs. 193.7 ± 82.3 ml/100 g/min, *P* = 0.04) and significantly lower CBV (206.4 ± 95.1 vs. 257.4 ± 89.1 ml/100 g, *P* = 0.009). However, Type-1 lesions didn't show a significant difference in perfusion measurements with peri-NAWM.

The DTI-derived mean FA and MD values as well as DSC-derived rCBF and rCBV values of three types of lesions and their comparisons (*P*-values) are summarized in Table 1. Compared to Type-1 lesions, Type-2 lesions showed significantly higher MD and lower FA. Compared to Type-1 lesions, Type-3 lesions only showed significant difference in MD (*P* = 0.036) but not in FA. Compared to Type-3 lesions, Type-2 lesions showed marginally higher MD and lower FA (*P* = 0.04). The mean rCBF and rCBV were the lowest in Type-2 lesions and were the highest in Type-3 lesions with Type 1 lesions being in the middle. The increased blood perfusion in Type-3 lesions may be associated with vascular inflammatory activities since most enhancing lesions (9 out of 11) were Type-3 lesions.

**Table 1:** Diffusion and perfusion imaging measurements in different types of MS lesions on SWI and their comparisons.

	**Individual lesion types**			**Comparison between types**		
**Imaging measurements**	**Type 1**	**Type 2**	**Type 3**	**Type 1 vs. Type 2**	**Type 1 vs. Type 3**	**Type 2 vs. Type 3**
	**hypointense lesions**	**Isointense lesions**	**hyperintense lesions**			
MD	1.13 ± 0.32	1.41 ± 0.03	1.27 ± 0.04	*P* < 0.0001	*P* = 0.036	*P* = 0.047
FA	0.31 ± 0.05	0.27 ± 0.07	0.30 ± 0.12	*P* = 0.0004	*P* = 0.792	*P* = 0.047
rCBF	1.05 ± 0.46	0.86 ± 0.34	1.39 ± 0.41	*P* = 0.036	*P* = 0.0003	*P* < 0.0001
rCBV	1.05 ± 0.42	0.86 ±0.41	1.67 ± 0.46	*P* = 0.039	*P* < 0.0001	*P* < 0.0001

*The values were reported in mean ± standard deviation. The unit for MD is in mm^2^/s. The reported rCBF and rCBV are relative (i.e., ratio) measurements and FA is an index for the amount of diffusion asymmetry between 0 and 1 within a voxel, therefore, they don't have absolute units. The intensity of different types of lesions is relative to the surrounding white matter on SWI*.

**Figure 3:**
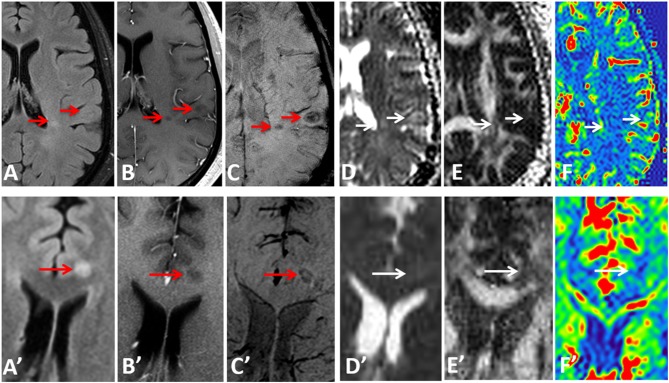
Representative images of Type-1 lesions in two MS patients (top row from a 36-year-old male patient and bottom row from a 37-year-old female patient) include FLAIR **(A,A′)**, Gd-enhanced T1-weighted **(B,B′)**, and SWI **(C,C′)**, as well as parameter maps of MD **(D,D′)**, FA **(E,E′)**, and CBF **(F,F′)**. The hypointense lesions on SWI (arrows) are associated with a less significant change in diffusion and perfusion measurements, as compared perilesional NAWM.

**Figure 4:**
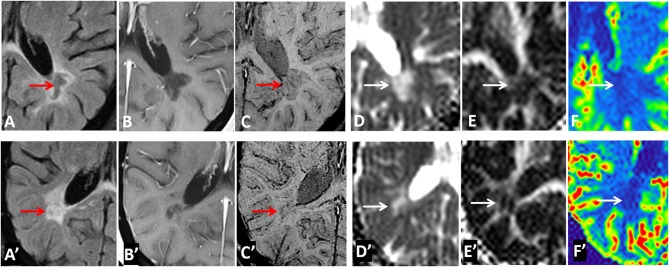
Representative images of Type-2 lesions in two MS patients (top row from a 43-year-old female patient and bottom row from a 37 female patient) include FLAIR **(A,A′)**, Gd-enhanced T1-weighted **(B,B′)**, and SWI **(C,C′)**, as well as parametric maps of MD **(D,D′)**, FA **(E,E′)**, and CBF **(F,F′)** The slightly hypointense (top row) or isointense (bottom row) lesion on SWI (arrow) showed a remarkable increase in MD, and decrease in FA and CBF, suggesting the chronic necrotic lesions indicated by the hypointensity on FLAIR, and T1-weighted imaging (i.e., top row) have severe microstructural destruction and disturbed perfusion.

**Figure 5:**
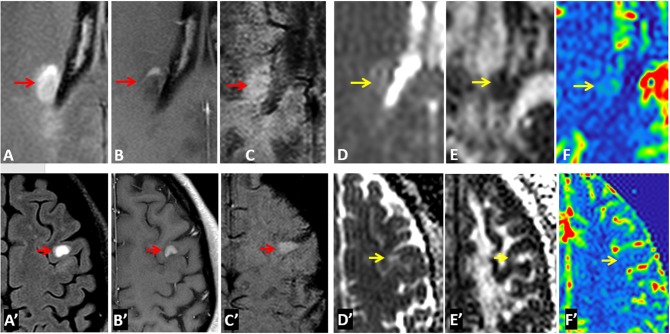
Representative images of Type-3 lesions in two MS patients (top row from a 32-year-old female and bottom row from 41-year-old female patient) include FLAIR **(A,A′)**, Gd-enhanced T1-weighted **(B,B′)**, SWI **(C,C′)**, as well as parametric maps of MD **(D,D′)**, FA **(E,E′)**, and CBF **(F,F′)**. The hyperintense lesions (arrow) on SWI showed gadolinium enhancement that is corresponding to increased perfusion and slightly increased MD as well as decreased FA.

**Figure 6:**
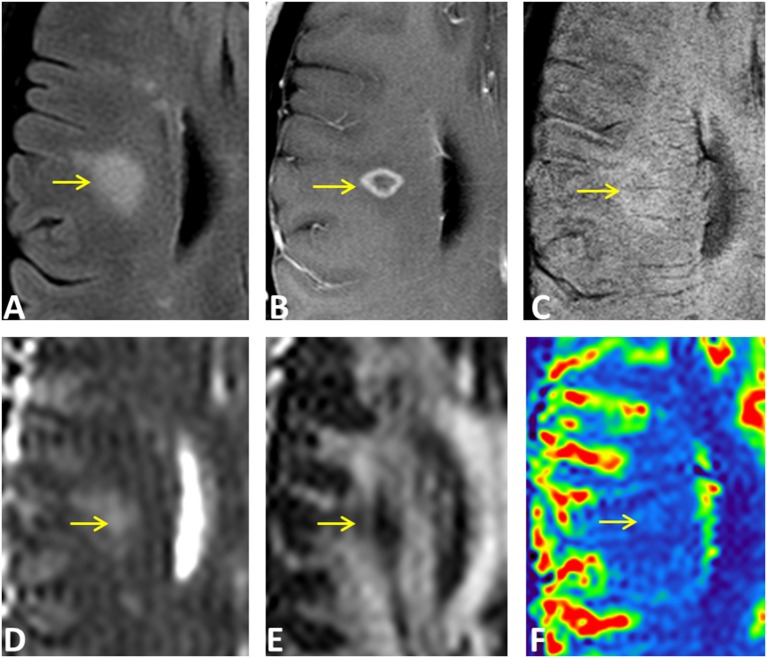
An ring-enhancing lesion in a 32-year-old female MS patient on FLAIR **(A)**, Gd-enhanced T1-weighted **(B)**, and SWI **(C)**, as well as on MD **(D)**, FA **(E)**, and rCBF **(F)** parametric maps. The lesion has both Type-2 (isointense center on SWI) and Type-3 (hyperintense rim on SWI) components. The center of the lesion demonstrated larger MD changes compared to the enhancing rim that has increased perfusion of the entire lesion.

Examples of diffusion and perfusion imaging parameter characteristics of Type-1 lesions were shown in Figure 3. As shown in one patient (in Figure 3 top row), SWI was most sensitive in detecting iron-laden component of lesions. The hypointense Type-1 lesions demonstrated a significant increase in MD and decrease in FA but no change in perfusion measurements compared to perilesional NAWM. Similarly, in another patient (Figure 3 bottom row), visible changes of MD and FA can be seen in another Type-1 lesion compared to peri-NAWM with uncertain perfusion changes. Representative Type-2 lesions were shown in Figure 4, in which SWI lesions that appeared as slightly hypointense (top row) or isointense (bottom row) showed a remarkable increase in MD and decrease in FA as well as reduced CBF. Such lesions in Figure 4 (top row) also showed hypointensity on both FLAIR and post-contrast T1-weighted images. Two Type-3 lesions with Gadolinium enhancement were shown in Figure 5, in which there is a mild increase in MD and marked decrease in FA as well as increase in CBF. One MS lesion with both Type-2 and Type-3 components was shown in Figure 6, in which the lesion showed a mixed pattern of significant diffusion and perfusion changes associated with non-enhancing center region and the enhancing rim, respectively.

## Discussion

Conventional MRI offers the most sensitive way to detect MS lesions and their changes over time for ruling in or ruling out a diagnosis of MS and for disease follow-up monitoring. The addition of SWI, which is a quick scan of routine conventional MRI protocols, may provide *in vivo* pathophysiological insights into cellular microstructural injury and tissue hemodynamic changes. Our results of MS lesions on SWI, combining quantitative multi-contrast and multi-parameter MRI, suggest that the intensity-based lesion types on SWI may represent a specific stage of lesion evolution or a certain pathological substrate associated with demyelination/axonal injury or inflammatory activity. These hidden pathological changes including blood-brain barrier dysfunction can possibly be detected with SWI without using Gadolinium contrast agent (26) as shown in Type-3 lesions. Our data also confirm previous imaging-histopathological correlative evidence of iron deposition, demyelination and axonal loss (6, 27, 28). In particular, three major observations emerge from these data. First, a hyperintense (Type-3) lesion on SWI may be related to the underlying enhanced vascular inflammatory activity with increased BBB disruption that results in increased CBF and CBV. Second, hypointense (Type-1) lesions on SWI are likely to have less tissue destruction by diffusion measures compared to Type-2 lesions; they also have less inflammatory activity than Type-3 lesions by perfusion measures. Third, isointense SWI Type-2 lesions may represent a more chronic demyelinated plaque with irreversible tissue destruction (e.g., black holes) showing the most severe DTI-derived diffusion changes.

SWI is a 3D gradient-echo high-resolution sequence that is fully flow-compensated with long-echo and combines magnitude and filtered-phase information to enhance susceptibility effects due to paramagnetic substances, such as hemosiderin and deoxyhemoglobin (10). Unlike quantitative susceptibility mapping (QSM), SWI is considered to be a qualitative MRI technique for enhanced lesion detection, its unique image contrast is particularly useful to gauge tissue iron content and venous structures. Therefore, it is well-recognized that hypointense (Type-1) lesions of MS are corresponding to increased iron content, which is likely due to chronic inflammatory activity with an elevated number of microglia and macrophages that contain high amounts of iron (15, 29). Type-1 lesions are also likely caused by increased hemosiderin (30) from old blood products leaked from inflammation-induced damaged vessels. All Type-1 lesions in this study were not enhancing on post-contrast T1-weighted imaging even though some lesions showed slightly increased blood perfusion, indicating certain inflammatory activities or lesion reactivity with increased macrophage cells (21). Compared to Type-2 lesions, these Type 1 lesions showed less diffusion abnormalities, which are believed to be corresponding to a lower degree of cellular architecture destruction during a tissue repair stage (8, 31).

Most SWI studies of MS have been focusing on hypointense (Type-1) lesions. In this study, besides the hypointense SWI lesions, we have also characterized quantitative diffusion and perfusion imaging features of isointense and hyperintense SWI lesions. Out of 137 MS lesions, 33.6% are Type-2 isointense lesions and 37.2% are Type-3 hyperintense lesions. Since mIP is a post-processed image using phase multiplication (with a factor of 4 in this study) and minimal intensity projection algorithm (10), the true meaning of signal intensity on multi-slice mIP images is uncertain. Therefore, in this study, the mIP image was generated using only one slice, in order to avoid the mixture effects of projected intensities. Except for high susceptibility substances (e.g., non-heme iron or venous blood) that contribute to dark signal on SWI, the non-dark signal on SWI is likely due to the combined effects of the amount free water content and edema (non-free intra- or extra-cellular water) due to pro-inflammation activation status. SWI does not provide a typical T1- or T2-weighted imaging contrast. After being applied with phase information, it does not seem to be a standard T2^*^ contrast either. Although the non-iron laden isointense or hyperintense SWI lesions have been consistently shown in the literatures and represent most MS lesions (16, 17, 32, 33), their histopathological characteristics are unclear. In this study, we found most isointense Type-2 lesions on SWI are corresponding to isointense or hypointense (or black hole) lesions on T1-weighted imaging. The well-demarcated black hole lesions on T1-weighted imaging likely represent the hypocellular area characterized by necrotic fluid elements and the loss of tissue structures.

Our results of combining quantitative diffusion and perfusion measurements support the notion that SWI can be used as a promising alternative in determining the underlying histopathological hallmarks of MS lesions. We found that Type-1 lesions have less diffusion changes than Type-2 lesions and less perfusion changes than Type-3 lesions, despite Type-1 lesions usually containing iron deposition. According to the previous study, the origins of iron deposition in MS lesions may be the concentrated iron of macrophages, debris of oligodendrocyte and myelin, or hemosiderin of hemorrhagic products from the leaky vessels (34, 35). The exact role of iron in MS is unclear with views from both sides that iron can either contribute to chronic inflammation, oxidative stress and neurodegeneration (35) or contribute to tissue repair (31). The slightly increased perfusion measurements (e.g., CBF and CBV) of Type-1 lesions found in this study may support the increased inflammation and cell activities.

To the best of our knowledge, this is the first time to characterize the hyperintense signal (Type-3) lesions on SWI with diffusion and perfusion measurements. Type-3 lesions showed significantly increased rCBF and rCBV but less diffusion changes compared to Type-2 lesions, indicating local vascular inflammation induced vasodilation and increased perfusion in these lesions (21, 36). This is also indicated by that fact that 9 out of 11 enhancing lesions in these patients are Type-3 lesions. Based on QSM analysis, Zhang et al. (26) showed gadolinium-enhancing MS lesions had relative low QSM values than non-enhancing lesions, which are consistent with the findings in this study that these enhancing lesions appear hyper- rather hypo-intense on SWI. Another study (32) has also demonstrated that enhancing lesions are likely to be hyperintensities in contrast to the central dark vein on post-gadolinium SWI images, despite that gadolinium is a paramagnetic agent and has strong T2^*^ shortening effect. These results suggest that signal intensities on SWI may help better detect BBB dysfunction and identify subtle inflammatory activities that are not detected on post-contrast T1-weighted imaging (35). The marginal or no difference of diffusion measurements between Type-3 and Type-1 lesions, as well as between Type-3 and Type-2 lesions, indicate that there is a large span of variabilities for microstructural changes in MS lesions depending on stages of lesion development and evolution. However, Type-2 lesions demonstrated the highest MD and lowest mean FA, suggesting most severe architecture destruction and tissue loss in these lesions; and Type-1 lesions showed the lowest mean MD, which may suggest a certain level of water diffusion restriction [i.e., cytotoxic edema from hypoxia injury (37, 38)] occurs in these lesions during high level of macrophage activities.

There were several limitations associated with this study. First, due to the challenge for quantifying the absolute CBF and CBV using DSC MRI (39, 40) due to the uncertainties of scaling coefficients for relaxivity and AIF partial volume, we used relative perfusion measures for comparison between lesion types. For comparisons between lesions and peri-NAWM, although we used the actual CBF and CBV values from DSC SVD algorithms, the values reported in these tissues are supposed to be interpreted for comparisons only. Future longitudinal studies are warranted for validating some of the findings regarding the underlying histopathology of lesion development and progression, in particular with a large sample size of patients with enhancing lesions. Lastly, the definition of different types of lesions was using 30% signal intensity difference may be arbitrary, however, we found the classification based on such a threshold provided appropriate differentiable imaging features from DTI and DSC data.

## Conclusion

This study indicates that the addition of SWI to clinical MRI protocol may provide *in vivo* pathological insights, suggesting that the intensity-based lesion types on SWI may represent a specific stage of lesion evolution or a certain pathological substrate associated with iron deposition, demyelination/axonal injury or inflammatory activity. Further studies investigating the longitudinal evolution of lesion appearances on SWI and their quantitative correlations will be envisioned.

## Data Availability

All datasets generated for this study are included in the manuscript and/or the supplementary files.

## Ethics Statement

This study was carried out in accordance with the recommendations of name of guidelines, name of committee; with written informed consent from all subjects. All subjects gave written informed consent in accordance with the Declaration of Helsinki. The protocol was approved by the local ethics committee of the Institutional Review Board Human Research Protection Program.

## Author Contributions

YG and HS participated in the design of this study, and they both performed the statistical analysis. HS collected important background information. BZ carried out literature search and interpretation of the data. All authors contributed to the construction of manuscript and its critical revision.

### Conflict of Interest Statement

The authors declare that the research was conducted in the absence of any commercial or financial relationships that could be construed as a potential conflict of interest.
